# Evaluation of Galectin-3 Plasma Concentration in Adolescents with Ventricular Arrhythmia

**DOI:** 10.3390/ijerph18052410

**Published:** 2021-03-02

**Authors:** Radosław Pietrzak, Tomasz M. Książczyk, Elżbieta Górska, Łukasz A. Małek, Bożena Werner

**Affiliations:** 1 Department of Pediatric Cardiology and General Pediatrics, Medical University of Warsaw, Żwirki i Wigury Street 63a, 02-091 Warsaw, Poland; tomasz.ksiazczyk@wum.edu.pl (T.M.K.); bozena.werner@wum.edu.pl (B.W.); 2 Department of Laboratory Diagnostics and Clinical Immunology of Developmental Age, Medical University of Warsaw, Żwirki i Wigury Street 63a, 02-091 Warsaw, Poland; elzbieta.gorska@wum.edu.pl; 3 Department of Epidemiology, Cardiovascular Disease Prevention and Health Promotion, National Institute of Cardiology, Alpejska Street 42, 04-628 Warsaw, Poland; lmalek@ikard.pl

**Keywords:** galectin-3, ventricular arrhythmia, adolescents

## Abstract

Galectin-3 (G3) is a biomarker known as an inflammatory state exponent. The aim of this paper was to analyze the G3 in adolescents with ventricular arrhythmia (VES) in order to evaluate its impact on myocardial tissue preservation. The study group (SG) consisted of 25 VES adolescents. The control group (CG) was 21 healthy children. G3 was assessed in the SG and CG. In the SG electrocardiography, Holter monitoring, echocardiography and CMR were performed. The G3 in SG was 13.45 ± 11.4 ng/mL and in CG 7.2 ± 2.0 ng/mL, *p* < 0.001. Moderate positive correlation between the G3 and z-score of the left ventricular diameter (r = 0.47, *p* = 0.041) and moderate negative correlation between the G3 and the left ventricular ejection fraction in cardiac magnetic resonance (CMR EF) (−0.49, *p* = 0.032) were found. According to the multiple linear regression analysis, CMR EF and VES were independent predictors for G3 elevation. Conclusion: Galectin-3 plasma concentration is elevated and correlates with the chosen left ventricular dysfunction parameters in adolescents suffering from ventricular arrhythmia. Further investigation is necessary to establish if elevated G3 is a useful biomarker for screening young individuals with ventricular arrhythmia who are at risk of structural cardiovascular pathology.

## 1. Introduction

Galectin-3 is a protein produced by many tissues and is involved in a variety of processes, such as inflammatory reaction, apoptosis, fibrosis, or heart remodeling. This biomarker is considered as a novel, useful tool in many clinical situations, and among them in the area of cardiology [1].

Galectin-3 plasma concentration is elevated in patients with persistent atrial fibrillation, and it is useful for the prediction of atrial fibrillation recurrence after catheter ablation [2,3,4,5,6]. This protein may be used as a predictor of mortality and morbidity in patients suffering from acute and chronic heart failure [7,8]. Moreover, there are some trials suggesting that galectin-3 may be regarded as a biomarker in the diagnosis of dilated cardiomyopathy, arrhythmogenic right ventricular cardiomyopathy, or left ventricular hypertrophy [9,10,11]. 

The prevalence of ventricular arrythmia in young populations is extremely common. It is present in 16–50% of children, but the number of extra beats usually does not exceed 50 per day in each individual [12]. A screening study in asymptomatic, school-age Japanese children reported the presence of ventricular arrhythmia in resting electrocardiography (ECG), in 2–8 per 100,000 children [13]. If the arrhythmia burden is not high (when assessed in 24 h Holter monitoring) and the heart morphology is normal, it is considered a benign phenomenon. However, in clinical practice we observe a “grey zone” of young active individuals in whom dyssynchronous contractile activity due to ventricular arrhythmia leads to borderline systolic function and slight enlargement of the left ventricle. In this clinical scenario, differential diagnosis between a benign, transient phenomenon and the disease is often difficult, all the more so because the exact number of arrhythmias that cause myocardial impairment is poorly evaluated in the literature. In light of these data, we assume that galectin-3 may be a novel tool in the assessment of ventricular arrhythmia in adolescents, when severe pathology of cardiovascular system is considered. 

The aim of this study was to analyze the galectine-3 plasma concentration in adolescents with ventricular arrhythmia in order to evaluate its impact on myocardial tissue preservation. 

## 2. Materials and Methods

The study group consisted of 25 consecutive children, aged 11–17 years, mean 14.7 years, diagnosed with ventricular arrhythmia. The lower limit for the diagnosis of ventricular arrhythmia was 100 extra ventricular beats per 24 h and/or complex arrhythmia detection. Complex arrhythmia was defined as it was described previously in the literature [14]. Arrhythmia was detected in the standard ECG, and/or 24 hours Holter monitoring, which were performed after initial diagnosis of irregular heart rhythm in the auscultation or as a screening procedure before participation in a leisure sport. Patients were referred to the Department of Cardiology for further assessment between March and October 2020. In all patients’ routine cardiological assessments, including detailed history of chronic diseases, 12-lead ECG, 24 h Holter monitoring, and echocardiography were performed, and a sample of blood was taken for the galectin-3 analysis. Subsequently, cardiac magnetic resonance was performed within one month of the first visit. 

The control group consisted of 21 healthy children aged 11–17 years, mean 14.6 years, matched to the study group according to the age and body size.

The study was approved by the Bioethical Committee of the Medical University (No. 98/2020).

### 2.1. Galectin-3

Plasma collections for galectin-3 concentration assessments were performed in all consecutive patients from the study group and in children from the control group. A sample of 2.7 mL of peripheral venous blood was drawn into EDTA-containing tubes, centrifuged immediately, and stored at −80 degreases Celsius. Frozen samples from the trial sites were stored for the analysis. Analysis of galectin-3 concentration was performed using an enzyme-linked immunosorbent assay (ELISA) developed by BioVendor Research and Diagnostic Products (BioVendor-Laboratorni medicina, a.s., Brno, Czech Republic). The lower limit of detection for this assay is 0.29 ng/mL, which does not cross-react with collagens, other members of the galectin family or common heart failure medications.

### 2.2. ECG and 24 h Holter Monitoring

Standard, resting 12-lead electrocardiograms were obtained. The morphology of ventricular extra beats was assessed, if present. Furthermore, arrhythmia was evaluated using 24 h Holter monitoring, where the number and complexity of arrhythmia were assessed.

### 2.3. Echocardiography

Adolescents in the study group were examined by an experienced pediatric cardiologist using a standardized protocol with an EPIQ ultrasound system (Philips). Procedure was performed with the patient being in the left lateral decubitus position with a phased-array transducer S5-1. Routine measurements of the left ventricular diameter (LVDd) and right ventricular outflow tract diameter (RVOTd) in parasternal long (PLAX) and short axis (PSAX), as well as left ventricular ejection fraction (LV EF) using the Teicholz method, were performed according to clinical practice and international guidelines [15]. Additionally, the RVOTd measurements were indexed to the body surface area (BSA), and LVDd was expressed as a z-score.

### 2.4. Cardiac Magnetic Resonance (CMR)

CMR scanning was performed with the use of a 3 Tesla Philips Achieva MR system (Philips Healthcare) as described earlier in the literature [16]. In particular, cine steady state free precession (SSFP), dark blood T2-weighted, and late gadolinium enhancement (LGE) images were acquired. Images were acquired within one month of the patient’s first visit. All CMR studies were analyzed offline using a dedicated software. Quantitative and qualitative data analyses were performed by an expert investigator blinded to clinical and ECG data. The following parameters were measured: left ventricular end diastolic volume, left ventricular end systolic volume, right ventricular end diastolic volume, and right ventricular end systolic volume, all of which were indexed to the body surface area. Left and right ventricular ejection fraction were also assessed.

### 2.5. Statistical Analysis

The Kolmogorov–Smirnov test was used to verify a normal distribution. In case of normal distribution data was presented as mean ± SD, whether with not normal distribution it was presented as median and range. Comparison between the controls and the study group was determined by using the Mann–Whitney U test. Correlations were determined according to the Spearman rank order correlation. Multiple linear regression analysis was made in order to assess galectin-3 plasma concentration on the basis of arrythmia number and ejection fraction calculated in the cardiac magnetic resonance. A receiver operating characteristic (ROC) curve was generated to determine the sensitivity and specificity of arrhythmia burden to detect increased galectin-3 plasma concentration. The statistical analysis was not performed if the number of patients was less than 10. A *p* < 0.05 was taken as being statistically significant.

## 3. Results

None of the patients suffered from any chronic diseases. The demographical data of the study and control group are summarized in Table 1.

### 3.1. Arrhythmia in the Study Group

Arrhythmia was detected in seven patients in the resting ECG. In six of them, ectopic beats had a morphology suggesting right ventricular outflow tract origin. In one patient, ectopic beat morphology suggested posterior wall origin of the arrhythmia. In the rest of the children, arrhythmia was registered in 24 hours Holter monitoring after initial diagnosis of irregular rhythm in physical examination. In the 24 hours Holter monitoring, the median number of beats was 5944 beats (range: 20–41,183 beats) and accounted for 7% (range: 0–46%) of arrhythmia burden. Fourteen patients were diagnosed with complex arrhythmia.

### 3.2. Galectin-3

**Figure 1:**
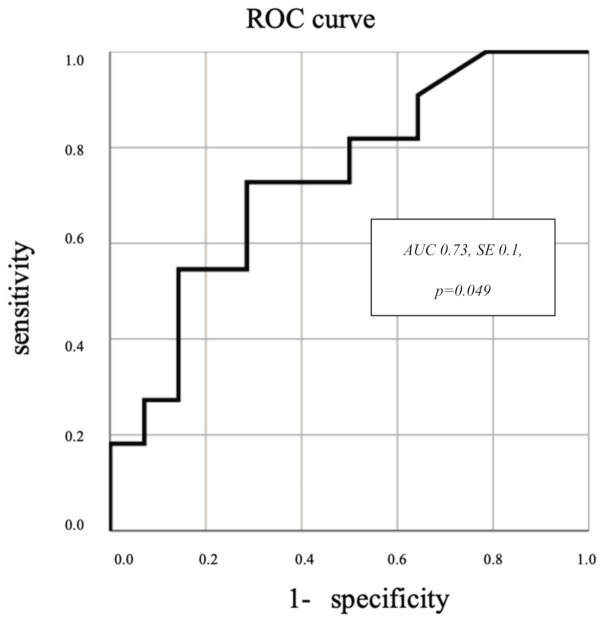
Receiver operating characteristic (ROC) curves for arrhythmia burden to detect cut-off value for increased galectin-3 plasma concentration. AUC—area under the curve, SE—standard error.

The plasma concentration of galectin-3 in patients in the study group was 13.45 ± 11.4 ng/mL. This was significantly higher (*p* < 0.001) compared to its level in children in the control group, for whom the value was 7.2 ± 2.0 ng/mL (Tables S1, S2 and Figure 1). Galectin-3 plasma concentration was not statistically significantly higher (*p* > 0.05) in children with complex arrhythmia (16.8 ± 14.2 ng/mL) compared to the protein level in patients without complex arrhythmia (9.1 ± 2.2 ng/mL) and in the control group (Table S3 and Figure 3).

### 3.3. Galectin-3 and Arrhythmia Burden

We analyzed ROC curves for arrhythmia burden to detect its cut-off value for increased galectin-3 plasma concentration. The elevated plasma concentration of galectin-3 was fixed at above 10.71 ng/mL (the maximal galectin-3 level in the control group) and was increased in eleven patients of the study group. According to the results of the ROC analysis, a statistically significant correlation (area under the curve (AUC) 0.73, SE 0.1, *p* = 0.049) was achieved, as shown in Figure 1. The optimal point of arrhythmia burden for galectin-3 elevation was 6972 beats with a sensitivity of 73% and specificity of 71%. A detailed table of the results is presented in the Supplementary Materials (Table S4).

### 3.4. Echocardiography and CMR

Echocardiography was performed in all patients and CMR in 15 patients. All echocardiographic data are summarized in Table 2 and the CMR parameters in Table 3. In the CMR, mid-wall fibrosis within the left ventricular muscle was diagnosed in one patient (galectin level 6.9 ng/mL).

Significant pathology of the circulatory system was diagnosed following the echocardiography and/or cardiac magnetic resonance in four patients, as presented in Table 4.

In a 14-year-old boy diagnosed with dilated cardiomyopathy, an extremely high percentage of arrhythmia burden (41.6%) was detected. Galectin-3 plasma concentration was 25.2 ng/mL. In that patient, left ventricular diastolic diameter was increased (z +3.6, when detected in echocardiography and 112 mL/m^2^ in CMR), and EF was as low as 55% in echocardiography and 51% when analyzed in CMR. A 16-year-old girl was diagnosed with arrhythmogenic ventricular cardiomyopathy on the basis of the following criteria: non-sustained ventricular tachycardia of left bundle-branch morphology with superior axis, inverted T waves in right precordial leads, enlarged right ventricular outflow tract (RVOT PSAX 22 mm/m^2^) in echocardiography, and right ventricular end-diastolic volume in CMR (108 mL/m^2^). In this case, the arrhythmia burden was 11% and the galectin level was 19.1 ng/mL.

In the other two patients, classical mitral valve prolapse was diagnosed. In both, non-sustained ventricular tachycardias were observed with galectin levels of 9.0 and 9.2 ng/mL.

### 3.5. Galectin-3 and Echocardiography Data

**Figure 2:**
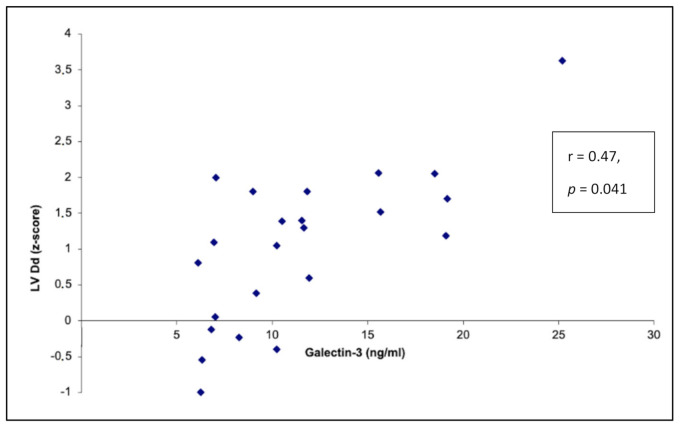
Correlation between galectin-3 plasma concentration and left ventricular diastolic diameter expressed as a z-score (r = 0.47, *p* = 0.041).

Correlations between galectin-3 plasma concentration and echocardiographic parameters are summarized in Table 5 and Table S5. The research demonstrated statistically significant moderate positive correlation between galectin-3 plasma concentration and left ventricular diastolic diameter expressed as a z-score (r = 0.47, *p* = 0.041) (Figure 2). Moderate negative correlation between galectin-3 plasma level and the ejection fraction was observed. However, it was not statistically significant. Galectin-3 plasma concentration did not correlate with the right ventricular outflow tract diameter parameters.

### 3.6. Galectin-3 and CMR Parameters

**Figure 3:**
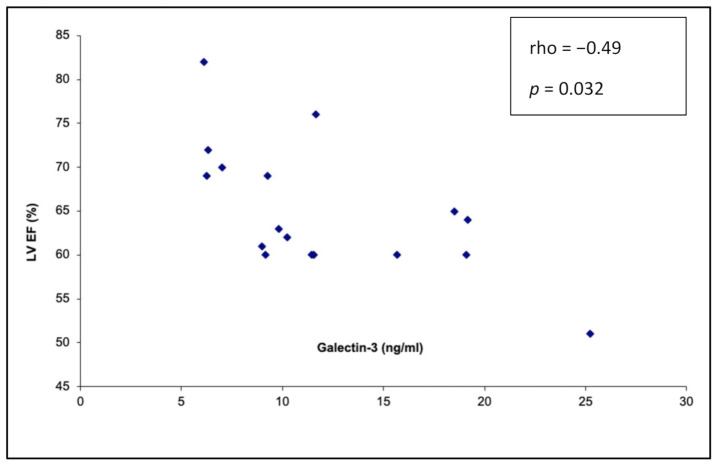
Correlation between the galectin-3 plasma concentration and left ventricular ejection fraction (%) measured in the cardiac magnetic resonance (r = −0.49, *p* = 0.032).

Correlations between galectin-3 plasma concentration and cardiac magnetic resonance parameters are summarized in Table 6 and Table S6. Statistically significant, moderate negative correlation between galectin-3 plasma concentration and the left ventricular ejection fraction (−0.492, *p* = 0.032) was detected (Figure 3). In the cardiac magnetic resonance, correlations between the galectin-3 level and systolic and diastolic ventricular volumes were not accomplished.

### 3.7. Multivariate Analysis

Multiple linear regression analysis considering evaluation of galectin-3 plasma concentration on the basis of arrythmia number and the left ventricular ejection fraction assessed in cardiac magnetic resonance was statistically significant (*F* (2, 21) = 7.29; *p* = 0.004.) The arrhythmia number is a slightly stronger predictor of galectin-3 plasma level than the left ventricular ejection fraction (Table 7 and Table S7).

## 4. Discussion

During a well-child examination, an irregular heart rhythm is often accidentally discovered in asymptomatic pediatric patients. In such a clinical scenario, ventricular ectopy is common and usually considered benign if the patient’s heart is structurally normal and clinical symptoms do not exist. On the contrary, a high burden of extra beats or ventricular arrhythmia that worsens with exercise accompanied by cardiac, morphological abnormalities raises suspicion of severe pathology, which could deteriorate the cardiac muscle [16,17]. Galectin-3 as a marker of myocardial impairment may indicate those patients with ventricular arrhythmias who are at risk of such clinical circumstances.

We discovered that galectin-3 plasma concentration is increased in adolescents with the ventricular extra beats when compared to healthy peers. To the best of our knowledge, galectin-3 has not yet been investigated in patients with ventricular arrhythmia. However, its significance in supraventricular heart rhythm disturbances, mainly in atrial fibrillation, is broadly described.

Galectin-3 expression levels are increased in patients with atrial fibrillation and, to some point, may be useful in the assessment of the treatment results. Its plasma concentration predicts arrhythmia recurrence following a single ablation procedure, whilst the data are conflicting when considering the value of galectin-3 in the anticipation of the ablation effectiveness. Furthermore, galectin-3 was independently associated with atrial remodeling in patients with chronic atrial fibrillation [3,4,5,18,19].

In our study, ROC analysis reveals statistically significant correlation (AUC 0.73, SE 0.1, *p* = 0.04) between the arrhythmia number and galectin-3 plasma level, which confirms the hypothesis that dyssynchronous contractile activity due to ventricular arrhythmia is the ground for myocardial degeneration. Unfortunately, the sensitivity (73%) and specificity (71%) of optimal cut of point are relatively low. Nevertheless, the data in the literature suggest that in some clinical situations, the value of galectin-3 measurement may be improved by analysis with other biomarkers such as NT-proBNP [20]. This raises hope that galectin-3 may be a valuable biomarker in detecting those patients who are at risk of early myocardial tissue impairment due to arrhythmia.

In our study group, there were two cases of significant pathology of the myocardium: dilated cardiomyopathy and arrhythmogenic right ventricular cardiomyopathy. In both cases, the first sign of the disease was a high burden of ventricular arrhythmia and increased galectin-3 plasma concentration.

In their trial in athletes, Biffi A et al. [17] empirically estimated that the number of extra beats—exceeding 2000 per day—may be related to severe pathologies of the circulatory system, such as hypertrophic and dilated cardiomyopathy or arrhythmogenic right ventricular cardiomyopathy. Analyzing our ROC curve, we assume that 2000 extra beats is linked to the high sensitivity and low specificity point in the evaluation of myocardial impairment. Nevertheless, undiagnosed cardiomyopathy is a major cause of sudden cardiac death during physical activity, so this approach is reasonable in adolescents who are usually physically active. The postulated mechanism of sudden cardiac death in cardiomyopathies is ventricular tachyarrhythmia due to inflammation and fibrotic replacement of the myocardial tissue. In our study, myocardial fibrosis was observed in only one patient in cardiac magnetic resonance but, according to the literature, galectin-3 is a marker of early damage before fibrosis appears [1,6,9]. Thus, galectin-3 seems to be unique as a screening marker for those patients who are at risk of life-threatening arrhythmic events before obvious pathology is detectable in the imaging.

Our study depicts a positive correlation between galectin-3 plasma concentration with the left ventricular diastolic diameter and a negative correlation with contractile function of the left ventricle. In the multiple linear regression analysis, the CMR left ventricular ejection fraction and arrhythmia number were independent predictors for galectin-3 elevation. Furthermore, after excluding two patients with diagnosed significant pathology of the circulatory system (dilated cardiomyopathy (DCM) and arrhythmogenic right ventricular radiomyopathy (ARVC)) from our cohort, a statistically significant (*p* = 0.009) difference of galectin-3 plasma concentration in the study and control group was still observed (Table S2). There are two possible explanations of our findings. Firstly, in this small population, there may be patients with “hidden” pathology, i.e., in the occult/initial phase of disease with a relatively large ventricle, suppressed contractile function and high galectin-3 plasma concentration, but who still do not meet criteria for the diagnosis of a cardiomyopathy. Secondly, dyssynchronous contraction of the left ventricle due to the high arrhythmia burden increases workload of the myocardial fibers, exceeding their capacity. This adverse hemodynamical status may lead to gradual myocardial tissue damage expressed as an increased galectin-3 plasma concentration.

The evidence for our assumptions does not yet exist in the literature. Nevertheless, in adults, a correlation between ventricular arrhythmia burden and left ventricular systolic dysfunction has been reported [21,22,23,24]. To some point, it is reversible, but if not recognized in time, it will lead to a clear clinical picture of heart failure.

Currently, galectin-3 is a valuable marker for the diagnosis of heart failure that precisely predicts increased risk for mortality during mid-term follow-up. In the clinical course of heart failure, plasma concentration elevates over time, and the increase is independently associated with a poorer clinical outcome [7,25,26]. Furthermore, it is a useful biomarker either in the initial or in the ultimate stage of the disease. According to Yao Y et al. [27], it is associated with left ventricular myocardial overload and, therefore, may be used for the detection of early cardiac remodeling in adult patients with hypertension. On the other hand, galectin-3 exhibits dynamic changes during mechanical unloading and predicts survival rate following the use of a left ventricular assist device. It is also associated with the development of cardiac allograft vasculopathy after heart transplantation [28].

Finally, although the ejection fraction correlates moderately with the galectin level in both echocardiography and cardiac magnetic resonance, we did not achieve statistical significance in the case of echocardiography. There are several reasons that may explain this difference. Firstly, the methodologies of ejection fraction assessment in cardiac magnetic resonance and echocardiography are different. In cardiac magnetic resonance ejection, fraction analysis is based on volume changes, while in echocardiography, it is performed after simple measurement of the diameter followed by a mathematical calculation based on the premise that the left ventricular shape is elliptic. During the echocardiographic study, every small alteration of the measurement is powered by the formula for the ejection fraction calculation, which creates a higher variability of the results, so that statistical significance may be accomplished only in a relatively large cohort of patients.

On the basis of our findings, we conclude that galectin-3 can be a useful tool in the screening of adolescents with ventricular arrhythmia. However, further trials are necessary to establish the extent to which it may be applied in everyday clinical practice.

Some limitations could also bias our results; e.g., stimulation of the cardiac beta-adrenoreceptors can affect the number and complexity of arrhythmia as well as galectin-3 plasma concentration [29]. In such clinical situations, analysis of heart rate variability and its influence on galectin-3 plasma concentration could be useful in the sympathetic tone assessment. The authors’ intention is to continue the study with a broader range of analyzed parameters including heart rate variability.

## 5. Conclusions

Galectin-3 plasma concentration is elevated and correlates with the chosen left ventricular function parameters in adolescents suffering from ventricular arrhythmia. Further investigation is necessary to establish if elevated galectin-3 plasma concentration is a useful biomarker for screening young individuals with ventricular arrhythmia who are at risk of structural cardiovascular system pathology.

## Figures and Tables

**Table 1:** Demographical data in the study and control group.

	Study Group N = 25	Control Group N = 21	*p*
Age (years) Mean (±SD)	14.3(±2.7)	14.4 (±2.6)	>0.5
Body weight(kg) Mean (±SD)	50.4 (±13.7)	52.4 (±13.8)	>0.5
Height (cm) Mean (±SD)	163 (±16)	166 (±17)	>0.5
BSA Mean (±SD)	1.7 (±0.6)	1.8 (±0.6)	>0.5

BSA—body surface area.

**Table 2:** Echocardiographic data in the study group.

Study Group	Mean ± SD
LVDd (mm)	48.3 ± 4.9
LVDd (z-score)	0.8 ± 1.1
LV EF (Teicholz%)	69 ± 6.5
PLAX RVOTd (mm)	23.1 ± 4.7
PLAX RVOTd/BSA (mm/m^2^)	14.3 ± 5.3
PSAX RVOTd (mm)	24.7 ± 4.5
PSAX RVOTd /BSA (mm/m^2^)	15.0 ± 5.5

LVDd—left ventricular diameter, LV EF—left ventricular ejection fraction, PLAX RVOT right ventricular outflow tract diameter measured in parasternal long axis, PSAX RVOTd—right ventricular outflow tract diameter measured in parasternal short axis, BSA—body surface area.

**Table 3:** Cardiac magnetic resonance data in the study group.

Study Group	Mean ± SD
LV EF (%)	68.95 ± 12.23
iLVES vol (mL/m^2^)	34.08 ± 11.72
iLVED vol (mL/m^2^)	90.41 ± 12.83
RVEF (%)	58.09 ± 45.63
iRVES vol (mL/m^2^)	40.09 ± 8.09
iRVED vol (mL/m^2^)	92.17 ± 11.57
LV EF (%)	68.95 ± 12.23
iLVES vol (mL/m^2^)	34.08 ± 11.72

LVEF—left ventricular ejection fraction, iLVES vol—left ventricular end systolic volume indexed to the body surface area, iLVED vol—left ventricular end diastolic volume indexed to the body surface area, RVEF—right ventricular ejection fraction, iRVES vol—right ventricular end systolic volume indexed to the body surface area, iRVED vol—right ventricular end diastolic volume indexed to the body surface area.

**Table 4:** Patients with diagnosed pathology of the circulatory system.

Patient/Gender (Age/Years)	Boy (14)	Girl (16)	Girl (16)	Boy (13)
Diagnosis	DCM	ARVC	MVP	MVP
Gal-3 (ng/mL)	25.2	19.1	9.0	9.2
VES burden (number/%)	41,384/41.6	9524/11	<1	<1
Electrocardiography	-	Neg.T-V1-V5	-	-
Complexity of arrhythmia	ns-VT	ns-VT	ns-VT	ns-VT
Echocardiography	LVDd: z score +3.6 EF 55%	PLAX RVOT 19 mm/m^2^ PSAX RVOT 22 mm/m^2^	Bi leaflet prolapse	Anterior leaflet prolapse
CMR	iLVED vol: 112 mL/m^2^ LVEF 51%	iRVED vol: 108 mL/m^2^ RVEF 45%	-	-

DCM-dilated cardiomyopathy, ARVC—arrhythmogenic right ventricular radiomyopathy, MVP—mitral valve prolapse, Gal-3—galectin-3, VES—ventricular extrasystole, ns-VT- non-sustained ventricular tachycardia, LVDd—left ventricular diameter, PLAX RVOT—right ventricular outflow tract diameter measured in parasternal long axis, PSAX RVOTd—right ventricular outflow tract diameter measured in parasternal short axis, LVEF—left ventricular ejection fraction, iLVED vol—left ventricular end diastolic volume indexed to the body surface area, RVEF—right ventricular ejection fraction, iRVED vol—right ventricular end diastolic volume indexed to the body surface area.

**Table 5:** Correlations between galectin-3 plasma concentration and echocardiographic parameters.

Galectin vs.	rho	*p*
LVDd (mm)	0.17	0.94
iLVDd (z-score)	0.47	0.041
EF (%)	−0.3	0.15
PLAX RVOTd (mm)	0.13	0.55
PLAX iRVOTd (mm/m^2^)	0.17	0.46
PSAX RVOTd (mm)	0.06	0.78
PSAX iRVOTd (mm/m^2^)	0.18	0.40

LVDd—Left ventricular diastolic diameter, iLVDd—left ventricular diastolic diameter indexed to the body surface area, EF—left ventricular ejection fraction, PLAX RVOT—right ventricular outflow tract diameter measured in parasternal long axis, PLAX iRVOT—right ventricular outflow tract diameter measured in parasternal long axis indexed to the body surface area, PSAX RVOTd—right ventricular outflow tract diameter measured in parasternal short axis, PSAX iRVOTd—right ventricular outflow tract diameter measured in parasternal short axis indexed to the body surface area.

**Table 6:** Correlations between galectin-3 plasma concentration and cardiac magnetic resonance parameters.

Galectin. vs.	rho	*p*
LV EF (%)	−0.49	0.03
iLVES vol (mL/m^2^)	0.34	0.28
iLV ED vol (mL/m^2^)	0.09	0.73
RVEF (%)	−0.11	0.98
iRVES vol (mL/m^2^)	0.24	0.47
iRVED vol (mL/m^2^)	0.17	0.58

LVEF—left ventricular ejection fraction, iLVES vol—left ventricular end systolic volume indexed to the body surface area, iLVED vol—left ventricular end diastolic volume indexed to the body surface area, RVEF—right ventricular ejection fraction, iRVES vol—right ventricular end systolic volume indexed to the body surface area, iRVED vol—right ventricular end diastolic volume indexed to the body surface area.

**Table 7:** Multiple linear regression analysis for the variables dependent on galectin-3.

	*B*	*SE*	*β*	*t*	*p*
(Constant)	56.46	21.41		2.64	0.015
Arrhythmia number	0.0005	0.0001	0.49	2.88	0.009
CMR LV EF	−0.68	0.30	−0.38	−2.26	0.035

*B*—unstandardized coefficients, *SE*—standard error, *β*—standardized coefficients, t—*t* test.

## Data Availability

Publicly available datasets were analyzed in this study. This data can be found here: https://drive.google.com/file/d/1fTZajlbKWufU2gV36XI_xfqEs4DR40bh/view?usp=sharing. Accessed date since 01.03.2021.

## Supplementary Materials

The following are available online at https://www.mdpi.com/1660-4601/18/5/2410/s1, Table S1: Gelctin-3 level in the control and study group, Table S2: Gelctin-3 level in the control and study group after excluding patients with significant pathology of the circulatory system, Table S3: Galectin-3 level in the study group on patients with and without complex arrhythmia and in the study group, Table S4: Coordinates of ROC curve, Table S5: Galectin vs echo, Table S6: Galectin and CMR, Table S7: Multiple linear regression analysis for the galectin-3 plasma concentration, Figure S1: Galectin-3 plasma concentration in the study and control group, Figure S2: Galectin-3 level in the study group in patients with and without complex arrhythmia and in the control group.
