# Supplementary material for: Evaluation of Galectin-3 Plasma Concentration in Adolescents with Ventricular Arrhythmia
Source: Int J Environ Res Public Health. 2021 Mar 2;18(5):2410. doi: 10.3390/ijerph18052410 (PMC7967785; doi:10.3390/ijerph18052410)
Supplement: Supplementary file 1 [file ijerph-18-02410-s001.zip › ijerph-1101638-supplementary.pdf]

Table 1. Gelctin-3 level in the control and study group

	Study group (n = 25)		Control group (n = 20)					
	M	*SD*	*M*	*SD*	*U*	*Z*	*p*	*r*
Galectin [ng/ml]	13,45	11,63	7,29	2,12	96,5	-3,51	<0,001	0,52

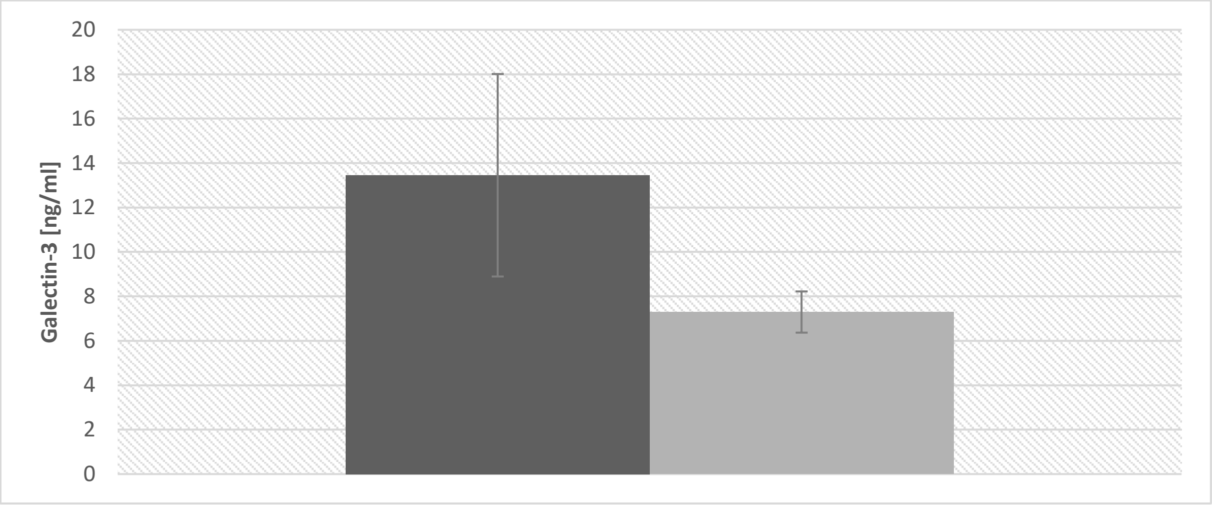

Figure 1. Galectin-3 plasma concentration in the study and control group.

Table 2. Gelctin-3 level in the control and study group after excluding patients with significant pathology of the circulatory system

	Study group (n = 23)		Control group (n = 20)				
	M	*SD*	*M*	*SD*	*U*	*Z*	*p*
Galectin [ng/ml]	12,69	11,12	7,29	2,12	600	2,62	0,009

Table 3. *Galectin-3 level in the study group on patients with and without complex arrhythmia and in the study group.*

	Witohut vomplex arrhythmia (n = 11)		Complex arrhythmia złożona (n = 14)		Control group (n = 20)			
	M	*SD*	*M*	*SD*	*M*	*SD*	*H*	*p*
Galectin [ng/ml]	9,15	2,30	16,83	14,75	7,29	2,12	14,84	0,001

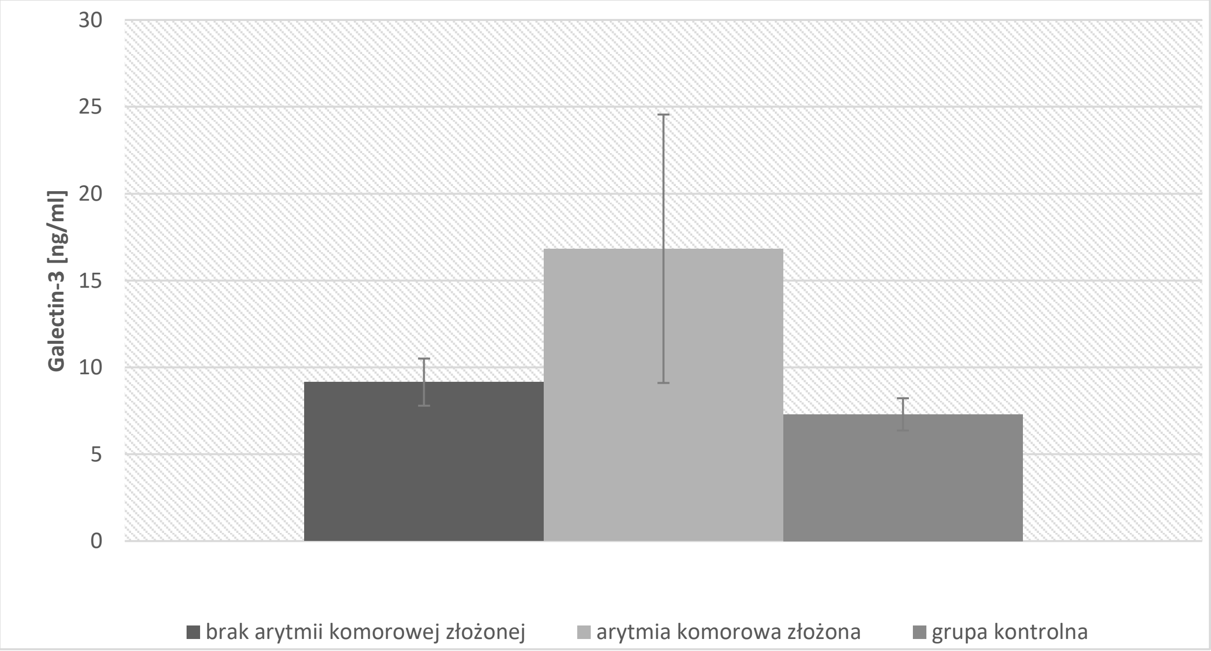

Figure 2. Galectin-3 level in the study group on patients with and without complex arrhythmia and in the study group.

Table 4 Coordinates of *ROC curve.*

Arrhythmia	Sensivity	1 - specifity
19	1	1
35	1	0,86
75	1	0,79
101	0,91	0,64
151	0,82	0,64
350	0,82	0,57
1370	0,82	0,50
2620	0,73	0,50
3327	0,73	0,43
4799	0,73	0,36
6972	0,73	0,29
8150	0,64	0,29
8900	0,55	0,29
9512	0,55	0,21
12262	0,55	0,14
15795	0,46	0,14
16795	0,36	0,14
18195	0,27	0,14
22195	0,27	0,07
25150	0,18	0,07
26168,5	0,18	0
34210,5	0,09	0
41385	0	0

Table 5

Galectin vs echo

		Galectin-3 [ng/ml]
LVDd	rho Spearmana	0,017
	p	0,936
Z	rho Spearmana	0,471
	p	0,041
EF	rho Spearmana	-0,304
	p	0,149
PLAX	rho Spearmana	0,132
	p	0,547
PLAX/m2	rho Spearmana	0,168
	p	0,456
PSAX	rho Spearmana	0,061
	p	0,782
PSAX m/2	rho Spearmana	0,183
	p	0,404

Table 6

Galectin and CMR

		Galectin-3 [ng/ml]
LV EF	rho Spearmana	-0,492
	p	0,032
LVES vol	rho Spearmana	-0,337
	p	0,284
LV EDvol	rho Spearmana	0,091
	p	0,729
RVEF	rho Spearmana	-0,11
	p	0,98
RV ESvol	rho Spearmana	0,242
	p	0,473
RV EDvol	rho Spearmana	0,175
	p	0,585

Table 7 Multiple linear regression analysis for the galectin-3 plasma concentration

	B	*SE*	β	*t*	*p*
(Constant)	56.46	21.41		2.64	.015
arrhythmia	0.0005	.0001	.49	2.88	.009
EF	-0.68	0.30	-.38	-2.26	.035
